# Safety of BCG vaccination and revaccination in healthcare workers

**DOI:** 10.1080/21645515.2023.2239088

**Published:** 2023-08-08

**Authors:** Paola Villanueva, Nigel W. Crawford, Mariana Garcia Croda, Simone Collopy, Bruno Araújo Jardim, Tyane de Almeida Pinto Jardim, Helen Marshall, Cristina Prat-Aymerich, Alice Sawka, Ketaki Sharma, Darren Troeman, Ushma Wadia, Adilia Warris, Nicholas Wood, Nicole L. Messina, Nigel Curtis, Laure F. Pittet

**Affiliations:** a Department of Paediatrics, The University of Melbourne, Parkville, VIC, Australia; b Infection and Immunity, Murdoch Children’s Research Institute, Parkville, VIC, Australia; c Infectious Diseases, Royal Children’s Hospital Melbourne, Parkville, VIC, Australia; d Department of General Medicine, Royal Children’s Hospital Melbourne, Parkville, VIC, Australia; e Immunisation Service, Royal Children’s Hospital Melbourne, Parkville, VIC, Australia; f School of Medicine, Federal University of Mato Grosso do Sul, Campo Grande, Brazil; g Department of Pediatrics, Universidade Estadual do Rio de Janeiro, Rio de Janeiro, Brazil; h Carlos Borborema Clinical Research Institute, Fundação de Medicina Tropical Dr. Heitor Vieira Dourado, Manaus, Brazil; i The University of Adelaide and the Women’s and Children’s Health Network, Adelaide, SA, Australia; j Julius Center for Health Sciences and Primary Care, University Medical Center Utrecht, Utrecht University, Utrecht, Netherlands; k Institut d’Investigació Germans Trias i Pujol, Departament de Genètica i Microbiologia, CIBER de Enfermedades Respiratorias (CIBERES), Instituto de Salud Carlos III, Universitat Autònoma de Barcelona, Badalona, Spain; l Department of Thoracic Medicine, Royal Adelaide Hospital, Adelaide, SA, Australia; m National Centre for Immunisation Research and Surveillance, Westmead, NSW, Australia; n The Children’s Hospital at Westmead, Westmead, NSW, Australia; o Wesfarmers Centre for Vaccines and Infectious Diseases, Telethon Kids Institute, Perth, WA, Australia; p Medical Research Council Centre for Medical Mycology, University of Exeter, Exeter, UK; q Department of Infectious Diseases, Great Ormond Street Hospital, London, UK; r The Children’s Hospital at Westmead Clinical School, Faculty of Medicine and Health, University of Sydney, Sydney, NSW, Australia; s Infectious Diseases Unit, Department of Paediatrics, Gynaecology and Obstetrics, Faculty of Medicine, University of Geneva, University Hospitals of Geneva, Geneva, Switzerland

**Keywords:** BCG, revaccination, vaccine adverse events, healthcare workers

## Abstract

BCG vaccination and revaccination are increasingly being considered for the protection of adolescents and adults against tuberculosis and, more broadly, for the off-target protective immunological effects against other infectious and noninfectious diseases. Within an international randomized controlled trial of BCG vaccination in healthcare workers (the BRACE trial), we evaluated the incidence of local and serious adverse events, as well as the impact of previous BCG vaccination on local injection site reactions (BCG revaccination). Prospectively collected data from 99% (5351/5393) of participants in Australia, Brazil, Spain, The Netherlands and the UK was available for analysis. Most BCG recipients experienced the expected self-limiting local injection site reactions (pain, tenderness, erythema, swelling). BCG injection site itch was an additional common initial local symptom reported in 49% of BCG recipients. Compared to BCG vaccination in BCG-naïve individuals, BCG revaccination was associated with increased frequency of mild injection site reactions, as well as earlier onset and shorter duration of erythema and swelling, which were generally self-limiting. Injection site abscess and regional lymphadenopathy were the most common adverse events and had a benign course. Self-resolution occurred within a month in 80% of abscess cases and 100% of lymphadenopathy cases. At a time when BCG is being increasingly considered for its off-target effects, our findings indicate that BCG vaccination and revaccination have an acceptable safety profile in adults.

## Introduction

Bacille Calmette-Guérin (BCG) vaccine is extensively and safely used in children in over 150 countries, to protect against tuberculosis (TB).^1^ BCG vaccination and revaccination are increasingly being considered for the protection of adolescents and adults against TB.^2,3^ There is also increasing interest in the broader applications of BCG vaccine for its beneficial ‘off-target’ immunological effects that protect against unrelated infectious^4–7^ and noninfectious diseases.^8^ However, the duration of off-target effects and the need for revaccination to maximize the benefits of BCG vaccine remain uncertain.

BRACE (BCG vaccination to reduce the impact of COVID-19 in healthcare workers) is a multicentre randomized controlled trial (ClinicalTrials.gov NCT04327206; date of registration 31/03/2020) that investigated whether BCG vaccination protects against coronavirus disease 2019 (Covid-19).^9^ We previously reported that BCG revaccination in Australian participants who were randomized within 3 days of influenza vaccination was associated with more frequent injection site abscess and regional lymphadenopathy.^10^ In this report, we evaluate the overall incidence of local adverse events and serious adverse events in the BRACE trial, as well as the impact of revaccination on local injection site reactions.

## Materials and methods

### Setting and participants

This prospective cohort study is nested within the BRACE trial, which recruited healthcare workers (HCW) in two stages. The trial protocol is described in detail elsewhere.^11^ In Stage 1, HCW were recruited in six hospitals in Australia from March to May 2020, and randomized in a 1:1 ratio and open-label design to receive BCG vaccine or no BCG. Participants in Stage 1 also received an intramuscular quadrivalent inactivated influenza vaccine to the contralateral arm within 3 days of randomization regardless of randomization group. In Stage 2, HCW were recruited from 25 healthcare centers (hospital or medical clinics) in Australia, Brazil, Spain, The Netherlands, and the UK from May 2020 to April 2021. They were randomized in a 1:1 ratio and blind design to receive BCG vaccine or placebo saline intradermal injection. Exclusion criteria for both stages comprised any contra-indication to BCG, including previous significant local BCG adverse reaction, immunosuppression or pregnancy at the time of vaccination. Previous BCG vaccination more than a year prior to enrollment, or previous history of positive tuberculin skin test (TST), were not exclusion criteria.

### Intervention

Participants randomized to BCG received a single dose of BCG-Denmark (AJ Vaccines, Copenhagen), 0.1 ml (corresponding to 2–8 × 10^5^ colony-forming units of *Mycobacterium bovis*, Danish strain 1331) intradermally in the upper arm, using a short (10 mm) bevel needle (25 G to 30 G). Participants randomized to placebo received a single dose of saline placebo intradermal injection, 0.1 ml intradermally in the upper arm, using a short (10 mm) bevel needle (25 G to 30 G). All participants were informed about the normal expected local reaction to BCG vaccination and were instructed to contact study staff if they had any concerns. If an individual previously had a BCG vaccine, the immunizers were instructed to administer the vaccine (BCG or placebo) a minimum of 2.5 cm from the original BCG scar.

### Data collection

Data were collected using REDCap web application^12^ including details on demographics, previous BCG vaccination, previous tuberculin skin tests (TST) and previous known latent tuberculosis infection (LTBI). Information on injection site evolution (including pain, tenderness, erythema, swelling), regional lymphadenopathy and serial vaccine site photographs (with ruler or standard coin for scale) were solicited through web-based daily questionnaires for 2 weeks following vaccination (vaccine diary) and a questionnaire at 3 months after vaccination. Participants could also contact the investigators by e-mail or telephone at any time after vaccination if they had any concerns about their injection site. As many participants spontaneously reported itch after vaccination, information on any vaccine site itch experienced within 3 months of vaccination was subsequently collected in the 6-month follow-up questionnaire.

Information on hospitalizations was collected through questionnaires at 3, 6, 9  and 12 months following vaccination. In the latter questionnaire, female participants were additionally asked regarding any pregnancy during the trial, to follow-up any participants who were inadvertently pregnant at the time of vaccination.

### Active safety surveillance

Designated safety medical doctors actively followed-up participants who reported a potential adverse event following immunization (AEFI) through the questionnaires or by notification of the study team. Reactions of grade 1 or 2 severity (as per the Food and Drug Administration (FDA) Toxicity Grading Scale for Healthy Adult and Adolescent Volunteers Enrolled in Preventive Vaccine Clinical Trials)^13^ were not considered ‘adverse,’ given that a normal BCG injection site reaction is characterized by a degree of pain, tenderness, erythema and swelling. Adverse events occurring within 3 months of vaccination were recorded on standard forms. They were classified as adverse events of special interest or serious adverse events (SAE).

Photographs of potential injection site abscesses, keloid scars, and unusual local reactions were reviewed by safety medical doctors at regular quality and safety team meetings for consensus decision on classification and whether any local clinical follow-up was required.

### Case definitions

BCG-revaccination was defined as BCG vaccination in a participant who had any prior BCG vaccination history. Adverse events of special interest included: injection site abscess, large ulcer (>1.5 cm diameter), keloid scar, unusual local reaction, regional lymphadenopathy, BCG osteitis/osteomyelitis, disseminated BCG infection (BCG-osis), allergic reaction due to vaccination or vasovagal episode following vaccination. Further details on case definitions are provided in Supplementary Material 1.

### Statistical analysis

StataIC 14.0 (Statacorp LP, College Station, TX, USA) was used for statistical analysis. The cumulative incidence of AEFI in the three-month post vaccination was calculated among participants who received either BCG or placebo, and who provided vaccine safety data. Local vaccination site reactions (pain, tenderness, erythema, swelling) were categorized according to the FDA toxicity grading scale^13^ and itch according to the Division of Acquired Immunodeficiency Syndrome (DAIDS) Table for Grading the Severity of Adult and Pediatric Adverse Events^14^ (see Supplementary Table S1). Local reaction grades at the vaccination site were compared in Stage 2 between participants who received BCG and those who received placebo, using Chi square or Fisher exact tests. Onset and duration of local reactions were compared using the Mann-Whitney test. Local reaction grades at the vaccination site were also compared between participants who were BCG-naïve at randomization and those who were BCG-revaccinated in Stage 1 and 2.

Ethical approval was obtained from The Royal Children’s Hospital Human Research Ethics Committee (HREC 62586) with subsequent approvals from all participating sites. All research was performed in accordance with relevant guidelines and regulations. All participants provided signed informed consent prior to enrollment.

## Results

### Demographics

**Figure 1.**
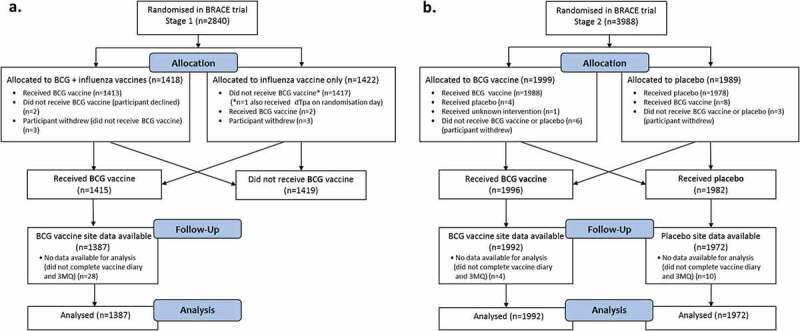
BRACE participants who received BCG or placebo in a) Stage 1 and b) Stage 2.

Abbreviations: BCG, Bacille Calmette-Guérin; dTpa, diphtheria-tetanus-acellular pertussis vaccine, reduced antigen formulation; 3MQ, 3-month questionnaire.

Among 3411 participants who received BCG (1415 in BRACE Stage 1 and 1996 in Stage 2) and 1982 participants who received placebo in Stage 2, 5351 (99%) provided vaccine safety data (Figure 1). Baseline characteristics are outlined in Table 1. Within BRACE Stage 2, these were similar between the BCG and placebo groups (Table 1). There was a higher proportion of BCG recipients with prior BCG vaccination history in BRACE Stage 2 compared with Stage 1 (77% versus 51%, respectively), as Stage 2 recruitment occurred predominantly in Brazil (a TB endemic country with BCG vaccination policy at birth). Most BCG-revaccinated participants (98%; 2200/2252) had received their last previous BCG vaccine more than 5 years prior.

**Table 1.** Demographics.

	Total *n* = 5351	BCG (Stage 1) *n* = 1387	BCG (Stage 2) *n* = 1992	Placebo (Stage 2) *n* = 1972
Sex – no. (%)				
Female	3976 (74)	1048 (76)	1443 (72)	1485 (75)
Male	1375 (26)	339 (24)	549 (28)	487 (25)
Age – years				
Median (IQR)	41 (32–51)	41 (31–51)	41 (32–51)	41 (32–51)
[range]	[18–83]	[18–73]	[18–78]	[18–83]
Country – no. (%)				
Australia	1804 (34)	1387 (100)	211 (11)	206 (10)
Brazil	2557 (48)	–	1283 (64)	1274 (65)
Netherlands	593 (11)	–	292 (15)	301 (15)
Spain	225 (4)	–	119 (6)	106 (5)
UK	172 (3)	–	87 (4)	85 (4)
Role – no. (%)				
Nurse/midwife	1334 (25)	569 (41)	396 (20)	369 (19)
Medical practitioner	664 (12)	261 (19)	212 (11)	191 (10)
Allied health worker	1083 (20)	230 (17)	420 (21)	433 (22)
Administrative/clerical	803 (15)	190 (14)	306 (15)	307 (15)
Scientist (medical/research)	219 (4)	45 (3)	86 (4)	88 (4)
PSA/hospital maintenance	716 (13)	78 (6)	326 (16)	312 (16)
Community health agent	187 (3)	–	87 (4)	100 (5)
Dentist/dental therapy	76 (1)	6 (<1)	29 (1)	41 (2)
Paramedic	57 (1)	–	28 (1)	29 (1)
Carer	41 (1)	–	20 (1)	21 (1)
Other	171 (3)	8 (<1)	82 (4)	81 (4)
Prior BCG history – no. (%)				
No	1584 (30)	673 (49)	454 (23)	457 (23)
Yes	3767 (70)	714 (51)	1538 (77)	1515 (77)
Lived in TB endemic country – no. (%)				
No	2479 (46)	1189 (86)	659 (33)	631 (32)
Yes	2834 (53)	172 (12)	1329 (67)	1333 (68)
Unknown	38 (<1)	26 (2)	4 (<1)	8 (<1)
Previous known LTBI – no. (%)				
No	5286 (99)	1357 (98)	1974 (99)	1955 (99)
Yes	37 (<1)	18 (<1)	11 (<1)	8 (<1)
Unknown	28 (<1)	12 (<1)	7 (<1)	9 (<1)
Previous TST – no. (%)				
Negative/none	4562 (85)	1032 (74)	1786 (90)	1744 (88)
Positive (>5 mm)	326 (6)	103 (7)	101 (5)	122 (6)
Unknown	463 (9)	252 (18)	105 (5)	106 (5)

Abbreviations: BCG, Bacille Calmette-Guérin; LTBI, latent tuberculosis infection; IQR, interquartile range; PSA, patient services assistant; TST, tuberculin skin test.

### Local injection site reactions

#### BCG versus placebo (Stage 2 participants only)

**Figure 2.**
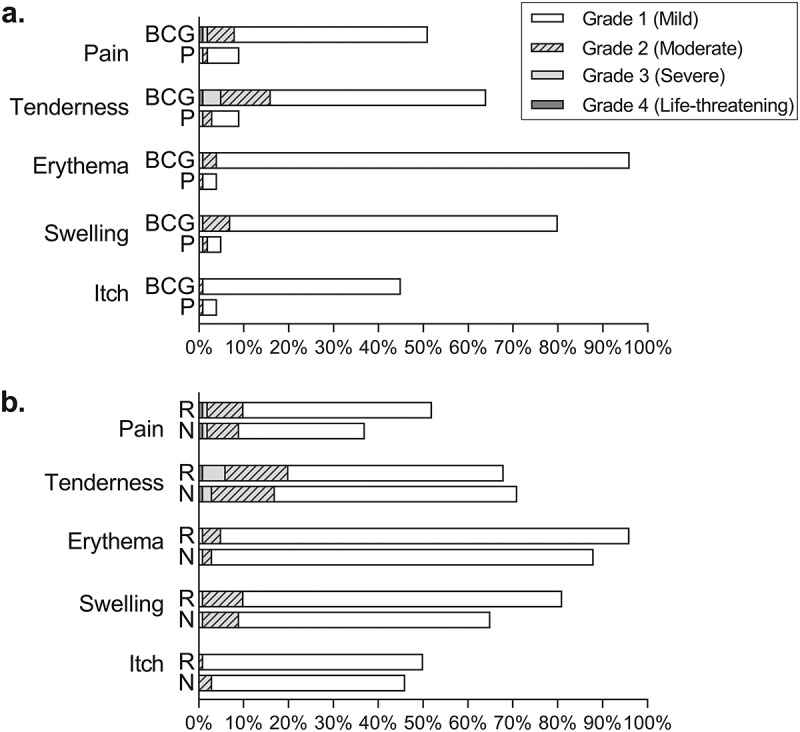
Local injection site reactions a) in Stage 2 participants and b) by prior BCG vaccination history, within participants receiving BCG in Stage 1 and 2.

Abbreviations: BCG, Bacille Calmette-Guérin; N, BCG-naïve; P, placebo; R, BCG-revaccination.

Overall, a local injection site reaction was reported in 1940/1992 (97%) BCG recipients and 331/1972 (17%) placebo recipients (*p* < 0.001); the majority of reactions were mild (grade 1) (Figure 2a).

A significantly higher proportion of participants in the BCG group compared with the placebo group, experienced pain (982/1992 [49.3%] versus 139/1972 [7.0%], *p* < 0.001), tenderness (1241/1992 [62.3%] versus 143/1972 [7.2%], *p* < 0.001), erythema (1878/1992 [94.3%] versus 66/1972 [3.3%], *p* < 0.001) or swelling (1589/1992 [79.8%] versus 64/1972 [3.3%], *p* < 0.001) at the injection site, and all reactions lasted longer (Table 2).

**Table 2.** Local injection site reactions for stage 2 participants.

	Total Stage 2	BCG	Placebo	*p*-value
	*n* = 3964	*n* = 1992	*n* = 1972	
**Pain**	1121 (28.3%)	982 (49.3%)	139 (7.0%)	<0.001
None	2843 (71.7%)	1010 (50.7%)	1833 (93.0%)	
Grade 1	991 (25.0%)	863 (43.3%)	128 (6.5%)	0.1^a^
Grade 2	122 (3.1%)	113 (5.7%)	9 (0.5%)	
Grade 3	7 (0.2%)	5 (0.3%)	2 (0.1%)	
Grade 4	1 (<0.1%)	1 (<0.1%)	0 (0.0%)	
Onset, days	2 (1–3)	2 (1–3)	1 (1–2)	<0.001
Duration, days	5 (2–9)	5 (3–9)	2 (1–4)	<0.001
**Tenderness**	1384 (34.9%)	1241 (62.3%)	143 (7.2%)	<0.001
None	2580 (65.1%)	751 (37.7%)	1829 (92.8%)	
Grade 1	1059 (26.7%)	947 (47.5%)	112 (5.7%)	0.04^a^
Grade 2	241 (6.1%)	212 (10.6%)	29 (1.5%)	
Grade 3	83 (2.1%)	81 (4.1%)	2 (0.1%)	
Grade 4	1 (<0.1%)	1 (0.1%)	0 (0.0%)	
Onset, days	2 (1–2)	2 (1–3)	1 (1–2)	<0.001
Duration, days	6 (4–12)	7 (4–13)	1 (1–3)	<0.001
**Erythema**	1944 (49.0%)	1878 (94.3%)	66 (3.3%)	<0.001
None	2020 (51.0%)	114 (5.7%)	1906 (96.7%)	
Grade 1	1891 (47.7%)	1826 (91.7%)	65 (3.3%)	1.0^a^
Grade 2	52 (1.3%)	51 (2.6%)	1 (0.1%)	
Grade 3	1 (<0.1%)	1 (0.1%)	0 (0.0%)	
Grade 4	0 (0.0%)	0 (0.0%)	0 (0.0%)	
Onset, days	1 (1–2)	1 (1–2)	1 (1–2)	0.03
Duration, days	13 (9–14)	13 (9–14)	2 (1–5)	<0.001
Maximal diameter, cm	1.5 (1.0, 2.0)	1.5 (1.0, 2.0)	0.5 (0.3, 1.0)	<0.001
**Swelling**	1653 (41.7%)	1589 (79.8%)	64 (3.3%)	<0.001
None	2311 (58.3%)	403 (20.2%)	1908 (96.7%)	
Grade 1	1522 (38.4%)	1460 (73.3%)	62 (3.1%)	0.04^a^
Grade 2	126 (3.2%)	125 (6.3%)	1 (0.1%)	
Grade 3	5 (0.1%)	4 (0.2%)	1 (0.1%)	
Grade 4	0 (0.0%)	0 (0.0%)	0 (0.0%)	
Onset, days	2 (1–3)	2 (1–3)	1 (1–2)	<0.001
Duration, days	9 (5–13)	9 (5–14)	1 (1–4)	<0.001
Maximal diameter, cm	1.0 (0.5, 1.5)	1.0 (0.5, 1.5)	0.5 (0.2, 1.0)	<0.001
	Total Stage 2 n = 3772	BCG n = 1900	Placebo n = 1872	
Itch*	926 (24.5%)	862 (45.4%)	64 (3.4%)	<0.001
None	2846 (75.5%)	1038 (54.6%)	1808 (96.6%)	
Grade 1	897 (23.8%)	839 (44.2%)	58 (3.1%)	0.01^a^
Grade 2	29 (0.8%)	23 (1.2%)	6 (0.3%)	
Grade 3	0 (0.0%)	0 (0.0%)	0 (0.0%)	
Grade 4	0 (0.0%)	0 (0.0%)	0 (0.0%)	
Onset, days	3 (2–7)	3 (2–7)	2 (1–6)	0.09
Duration, days	7 (4–20)	10 (5–20)	3 (2–7)	<0.001

Data are presented as n (%) or median (interquartile range), unless otherwise specified.

*Itch evaluated in 6-month follow-up questionnaire.

In the BCG group, antihistamines were taken by 13 participants, 1 participant applied a topical steroid cream and 1 took paracetamol to relieve the itch. In the placebo group, 2 participants took antihistamines and 1 participant took acyclovir for the itch.

^a^*p*-value comparing reaction severity Grades 1 to 4, between BCG and placebo groups.

#### Itch

A significantly higher proportion of participants in the BCG group experienced itching at the vaccination site, compared with the placebo group (862/1900 [45.4%] versus 64/1872 [3.4%], *p* < 0.001; Table 2). Itch duration was longer in the BCG group compared with the placebo group (median 10 days [interquartile range (IQR) 5–20] versus 3 days [IQR 2–7], *p* < 0.001). The majority 846 (98%) of BCG recipients did not take any medications for the itch. Antihistamines were not part of the study protocol, but were taken by 13 (<2%) participants (including six participants for 48 hours or longer) and one participant applied a topical steroid cream. In the placebo group, two participants took antihistamines for less than 48 h.

#### BCG-revaccination versus BCG-naïve

Among all BCG recipients in the BRACE trial, a local injection site reaction was reported in 1052/1127 (93%) BCG-naïve participants and 2195/2252 (97%) BCG-revaccinated participants (*p* < 0.001).

Revaccination compared with primary vaccination was associated with increased frequency, of pain, erythema, swelling, and itching (Table 3; Figure 2b). In contrast, tenderness was slightly less common but more severe in those who reported it. Swelling and erythema occurred earlier and for a shorter duration in the BCG-revaccinated group. In contrast, pain lasted longer (Table 4).

**Table 3.** Local BCG injection site reactions, by prior BCG vaccination history.

	Total BCG (Stage 1 & 2)	BCG-naïve	BCG-revaccination	p-value
	n = 3379	n = 1127	n = 2252	
**Pain**	1549 (45.8%)	399 (35.4%)	1150 (51.0%)	<0.001
None	1830 (54.2%)	728 (64.6%)	1102 (49.0%)	
Grade 1	1262 (37.4%)	312 (27.7%)	950 (42.1%)	0.2^a^
Grade 2	268 (7.9%)	82 (7.3%)	186 (8.3%)	
Grade 3	16 (0.5%)	4 (0.4%)	12 (0.5%)	
Grade 4	3 (0.1%)	1 (0.1%)	2 (0.1%)	
Onset, days	2 (1–3)	2 (1–3)	2 (1–3)	0.5
Duration, days	5 (2–9)	3 (2–9)	5 (3–9)	<0.001
**Tenderness**	2312 (68.4%)	797 (70.6%)	1515 (67.3%)	0.04
None	1067 (31.6%)	330 (29.3%)	737 (32.7%)	
Grade 1	1686 (49.9%)	610 (54.1%)	1076 (47.8%)	<0.001^a^
Grade 2	484 (14.3%)	161 (14.3%)	323 (14.3%)	
Grade 3	139 (4.1%)	25 (2.2%)	114 (5.1%)	
Grade 4	3 (0.1%)	1 (0.1%)	2 (0.1%)	
Onset, days	2 (1–3)	2 (1–3)	2 (1–2)	0.8
Duration, days	8 (4–14)	7 (3–14)	8 (4–13)	0.5
**Erythema**	3101 (91.7%)	976 (86.6%)	2125 (94.4%)	<0.001
None	278 (8.3%)	151 (13.4%)	127 (5.6%)	
Grade 1	2997 (88.7%)	954 (84.7%)	2043 (90.7%)	0.05^a^
Grade 2	99 (2.9%)	21 (1.9%)	78 (3.5%)	
Grade 3	5 (0.2%)	1 (0.1%)	4 (0.2%)	
Grade 4	0 (0.0%)	0 (0.0%)	0 (0.0%)	
Onset, days	2 (1–2)	2 (1–2)	2 (1–2)	<0.001
Mean [SD]	2.1 [2.4]	2.5 [3.3]	2.0 [1.8]	
Duration, days	13 (10–14)	14 (10–30)	13 (10–14)	<0.001
Mean [SD]	22.2 [26.5]	28.4 [31.1]	19.4 [23.6]	
Maximal diameter, cm	1.5 (1.0, 2.0)	1.0 (1.0, 2.0)	1.5 (1.0, 2.4)	<0.001
Mean [SD]	1.9 [1.4]	1.7 [1.4]	2.0 [1.4]	
**Swelling**	2539 (75.1%)	725 (64.3%)	1814 (80.5%)	<0.001
None	840 (24.9%)	402 (35.7%)	438 (19.5%)	
Grade 1	2243 (66.3%)	635 (56.3%)	1608 (71.4%)	0.6^a^
Grade 2	282 (8.3%)	87 (7.7%)	195 (8.7%)	
Grade 3	14 (0.4%)	3 (0.3%)	11 (0.5%)	
Grade 4	0 (0.0%)	0 (0.0%)	0 (0.0%)	
Onset, days	2 (1–3)	2 (1–3)	2 (1–3)	<0.001
Mean [SD]	2.8 [3.5]	3.3 [3.6]	2.7 [3.4]	
Duration, days	10 (5–14)	11 (4–21)	9 (5–13)	<0.001
Mean [SD]	14.1 [17.6]	18.8 [22.6]	12.3 [14.7]	
Maximal diameter, cm	1.0 (0.5, 2.0)	1.0 (0.7, 2.0)	1.0 (0.5, 2.0)	0.5
	Total BCG (Stage 1 & 2)	BCG-naïve	BCG-revaccination	
	n = 3122	n = 1018	n**=**2104	
Itch*	1535 (49.2%)	472 (46.4%)	1063 (50.5%)	
None	1587 (50.8%)	546 (53.6%)	1041 (49.5%)	
Grade 1	1474 (47.2%)	441 (43.2%)	1033 (49.1%)	0.001^a^
Grade 2	61 (2.0%)	31 (3.1%)	30 (1.4%)	
Grade 3	0 (0.0%)	0 (0.0%)	0 (0.0%)	
Grade 4	0 (0.0%)	0 (0.0%)	0 (0.0%)	
Onset, days	3 (2–7)	4 (2–7)	3 (2–7)	0.2
Duration, days	10 (5–20)	10 (5–15)	10 (5–20)	0.5

BCG recipients in Stage 1 and Stage 2. Data are presented as n (%) or median (interquartile range), unless otherwise specified.

*Itch evaluated in 6-month follow-up questionnaire.

^a^p-value comparing reaction severity Grades 1 to 4, between BCG-naïve and BCG-revaccination groups.

**Table 4.** Summary of local adverse reactions at BCG vaccination site in BCG-revaccinated compared with BCG-naïve group.

BCG-revaccinated vs BCG-naïve	Frequency	Severity	Size	Time to onset	Duration
Pain	Increased				Increased
Tenderness	Reduced	Increased			
Erythema	Increased		Increased	Reduced	Reduced
Swelling	Increased			Reduced	Reduced
Itch	Increased	Reduced			

### Adverse events of special interest

Of all BCG recipients, the most common adverse events of special interest were injection site abscess (55/3379, 1.6%) and regional lymphadenopathy (101/3379, 3%) (Table 5). In the placebo group, there were no injection site abscesses and 0.5% (9/1972) of participants reported regional lymphadenopathy. Overall, there were no cases of suppurative lymphadenitis.

**Table 5.** Adverse events of special interest.

	Total BCG (Stage 1 & 2) *n* = 3379	BCG (Stage 1) *n* = 1387	BCG (Stage 2) *n* = 1992	Placebo *n* = 1972
Injection site abscess	55 (1.6%)	41 (3.0%)	14 (0.7%)	0 (0.0%)
Large ulcer (>1.5 cm)	0 (0.0%)	0 (0.0%)	0 (0.0%)	0 (0.0%)
Keloid scar	2 (0.06%)	0 (0.0%)	2 (0.1%)	0 (0.0%)
Unusual local reaction*	4 (0.1%)	4 (0.3%)	0 (0.0%)	1 (0.05%)
Regional lymphadenopathy (axillary/neck)	101 (3.0%)	48 (3.5%)	53 (2.7%)	9 (0.5%)
Allergic reaction due to BCG**	1 (0.03%)	0 (0.0%)	1 (0.05%)	–
Vasovagal episode following vaccination^†^	3 (0.1%)	2 (0.1%)	1 (0.05%)	0 (0.0%)
BCG osteitis/osteomyelitis	0 (0.0%)	0 (0.0%)	0 (0.0%)	–
Disseminated BCG infection (BCG-osis)	0 (0.0%)	0 (0.0%)	0 (0.0%)	–

*BCG: 2 participants had a persistent firm swelling at the BCG site, without meeting abscess criteria; one surgically removed by local medical practitioner with local anaesthetic four months post vaccination, and the other (onset day 35 following vaccination, size 3 cm diameter) self-resolved after three months. One participant had a painful shoulder with restricted movement (onset day 51 following vaccination) self-resolving within one week. One participant had a diagnosis of disseminated granuloma annulare, commencing two months following vaccination, with a single buttock lesion.

*Placebo: 1 participant with intermittent tingling from left shoulder to elbow (onset day 30 following vaccination) self-resolving after 2–3 weeks.

**Participant with localized urticaria in left arm progressing to generalized pruritis, onset day 1, treated with antihistamines, resolved within one week.

^†^All occurred following blood sampling and receiving vaccination in patients with history of needle-associated vasovagal episodes.

#### Injection site abscess

The median time of onset was 20 days (IQR 9–26), with a median diameter of 2.0 cm (IQR 2.0–2.5) (Table 6). Two participants presented to an emergency department with severe injection site pain; one of these also had lethargy and was hospitalized for intravenous antibiotics and further investigations. This was classified as a ‘suspected unexpected serious adverse reaction (SUSAR)’ (as BCG abscess alone would not be expected to lead to hospitalization or systemic symptoms), which resolved after 2 weeks, following abscess discharge. Three participants, with 4.0 cm, 2.5 cm or 2.0 cm abscess each, had associated axillary lymphadenopathy. No other adverse events of special interest occurred concomitantly.

**Table 6.** Clinical features of BCG local adverse reactions (abscess and lymphadenopathy).

Injection site abscess	Total BCG	BCG (Stage 1)	BCG (Stage 2)	Placebo (Stage 2)
	*n* = 55	*n* = 41	*n* = 14	*n* = 0
Clinical features				
Time to onset, days	20 (2–45)	20 (3–45)	17 (2–38)	–
Maximum size, cm	2.0 (1.5–5.0)	2.0 (1.5–5.0)	2.0 (1.5–3.0)	
Abscess with discharge, No. (%)	54 (98%)	40 (98%)	14 (100%)	
Abscess with persistent discharge (>2w), No. (%)	32 (58%)	24 (59%)	8 (57%)	
Abscess with pain/tenderness at site, No. (%)	54 (98%)	40 (98%)	14 (100%)	
Management				
Observation, No. (%)	44 (80%)	34 (83%)	10 (71%)	–
Maximum size, cm	2.0 (1.5–5.0)	2.0 (1.5–5.0)	2.0 (1.5–3.0)	
Time to resolution, days	27 (2–243)	27 (2–243)	27 (4–102)	
Antimicrobial only, No. (%)	7 (13%)	5 (12%)	2 (14%)	
Topical antibiotic (mupirocin)	1	1	0	
Maximum size, cm	2.5	2.5	–	
Time to resolution, days	28	28	–	
Oral antibiotics (cephalexin/flucloxacillin)	3	2	1	
Maximum size, cm	3.0 (2.0–5.0)	3.5 (2.0–5.0)	3.0	
Time to resolution, days	21 (8–88)	15 (8–21)	88	
IV antibiotics (flucloxacillin)	1	0	1	
Maximum size, cm	nr	–	nr	
Time to resolution, days	16	–	16	
Oral isoniazid	2	2	0	
Maximum size, cm	4.3 (4.0–4.5)	4.3 (4.0–4.5)	–	
Time to resolution, days	131 (113–149)	131 (113–149)	–	
FNA + cephalexin/flucloxacillin, No. (%)	3 (5%)	2 (5%)	1 (7%)	
Maximum size, cm	2.0 (2.0–2.5)	2.0 (2.0–2.0)	2.5	
Time to resolution, days	30 (20–56)	43 (30–56)	20	
Surgical excision + co-amoxiclav, No. (%)	1 (2%)	0	1 (7%)	
Maximum size, cm	1.5	–	1.5	
Time to resolution, days	62	–	62	
Lymphadenopathy	Total BCG	BCG (Stage 1)	BCG (Stage 2)	Placebo (Stage 2)
	n = 101	n = 48	n = 53	n = 9
Clinical features				
Time to onset, days	5 (1–56)	6 (1–56)	4 (1–40)	4 (2–30)
Maximum size, cm	1.0 (0.5–4.0)	1.8 (0.5–4.0)	1.0 (0.5–4.0)	1.0 (0.5–5.0)
No. (%) with pain/tenderness at site	43 (43%)*	30 (63%)	13 (25%)	3 (33%)
Time to resolution, days	3 (1–30)	4 (1–30)	3 (1–14)	4 (1–22)

Abbreviations: FNA, fine needle aspiration; nr, not reported.

*Two participants took opioid analgesia for axillary pain associated with lymphadenopathy; one took codeine for two days and the other buprenorphine for four days following an emergency department presentation for the axillary pain.

Categorical variables are reported as number (%), continuous variables are reported as median (range).

The safety medical doctors recommended a conservative approach for all abscesses, except in two participants who were referred to an Infectious Diseases specialist due to persistent large abscesses (average size 4.3 cm diameter) and subsequently received 3 months of isoniazid. Nine other participants sought advice from external providers, who prescribed antimicrobial treatment, including three who additionally underwent fine needle aspiration and one who had surgical excision of the injection site. The other 44/55 (80%) of injection site abscesses resolved spontaneously without treatment, in a median time of 27 days (IQR 10–45).

#### Regional lymphadenopathy

Location of BCG-associated lymphadenopathy was ipsilateral axillary (*n* = 61, 60%), axillary and cervical (*n* = 11, 11%), axillary and supraclavicular (*n* = 1, 1%), cervical (*n* = 22, 22%), submandibular (*n* = 2, 2%), or supraclavicular (*n* = 4, 4%). The median time to onset was 5 days (IQR 3–8) following vaccination, with a median diameter of 1.0 cm (IQR 1.0–2.0) (Table 6). Overlying tenderness was experienced by 43/101 (43%) participants, but none had overlying erythema. One participant was treated with isoniazid for a concomitant injection site abscess of 4.0 cm diameter. Two participants took opioid analgesia for axillary pain associated with lymphadenopathy. All self-resolved, in a median time of 3 days (IQR 2–7).

Among the placebo group, regional lymphadenopathy was ipsilateral axillary (*n* = 5, 56%), or cervical (*n* = 4, 44%). One participant with persistent axillary lymphadenopathy (total duration 22 days) received oral cephalexin. The rest self-resolved in a median time of 4 days (IQR 3–6).

#### Other adverse events of special interest

Keloid scars occurred in 2/3379 (0.06%) BCG recipients and BCG-related allergic reaction in 1/3379 (0.03%) within the study period (Table 5). The allergic reaction, a case of localized arm urticaria on day of vaccination progressing to generalized pruritus, was managed with oral antihistamines and resolved within 1 week. There were no cases of large ulcers (>1.5 cm), BCG osteitis/osteomyelitis nor disseminated BCG infection.

### Serious adverse events

Overall, 38 participants reported a serious adverse event (SAE): 9 BCG recipients in Stage 1, 29 participants in Stage 2 including 20 in the BCG group and 9 in the placebo group (see Supplementary Table S2). All but two SAE were deemed ‘unrelated’ to the intervention by the study site investigator and the BRACE expert vaccine safety group (Table S1). One SAE (hospitalization in a participant with BCG injection site abscess and lethargy) was reported as a SUSAR, as aforementioned. Another BCG recipient was hospitalized and diagnosed with Crohn’s disease more than 2 months following vaccination, subsequently deemed ‘unlikely related’ to vaccination. There was one death (COVID-19 related) in a participant hospitalized with COVID-19 who had received placebo.

### Vaccination during pregnancy

Four female participants were vaccinated whilst unknowingly pregnant at the time; one of these received the BCG vaccine at gestational age of 2 weeks and the other three placebo (see Supplementary Table S3). There were no congenital anomalies or birth defects. All pregnancies resulted in healthy babies born at term gestation.

## Discussion

Using active safety surveillance, we evaluated the safety of BCG vaccination and revaccination in over 5000 healthcare workers within a large international trial. The majority of BCG recipients experienced the expected normal well-described local injection site reactions characterized by the appearance of a small, red papule or swelling at the injection site within 2–3 weeks. Usually, the papule softens, resulting in a small ulcer, healing over several weeks to months into a small flat scar.^15,16^

In a study of BCG-vaccinated infants in Guinea-Bissau, those who developed a BCG skin reaction by age 2 months had associated better survival, correlating with reaction size.^17^ Failure to develop a local BCG reaction, reported to occur in up to 10% of vaccinees, has been associated with incorrect administration technique, BCG strain type, vaccine batch or lack of immune response.^17–22^ Although the cause for the lack of initial local BCG reaction in a minority of BRACE trial participants is unclear, a subsequent study of BCG scar prevalence at 12 months following vaccination suggests that vaccine administration technique, prior BCG, study site, participant sex and age at vaccination are important factors.^23^

Itch at the BCG injection site was reported by half of BCG recipients with a median duration of 10 days following vaccination. This symptom has not been previously reported in relation to the BCG vaccine,^24^ likely due to the difficulty in assessing for the presence of this symptom in infants, to whom the vaccine is more commonly given. Self-limiting itch at the injection site has been reported following other vaccinations;^25^ postulated to be a symptom of post-injection inflammation, predominantly due to the host immune response to the vaccine components or the injection needle entering the skin.^26^ Needle injection depth also influences reactogenicity,^27^ as shown by other intradermal vaccines (such as influenza^28^ or COVID-19 vaccines^29^) being associated with increased injection site itch compared with the same vaccines given by the intramuscular route.

Revaccination has been associated with an increased risk of common local injection site reactions, as well as adverse events, such as injection site abscess and lymphadenopathy.^10,30^ In this study, which included participants from five countries, we found that a higher proportion of participants in the BCG-revaccinated group experienced the common local injection site reactions of pain, erythema, swelling, or itch, compared with the BCG-naïve group. Interestingly, injection site erythema and swelling occurred earlier and were of shorter duration in the BCG-revaccinated group. This may suggest the accelerated local BCG reaction phenomenon (accelerated onset and healing), which has been described in association with current or prior mycobacterial exposure, including BCG revaccination.^31–33^ An accelerated BCG reaction in children has been previously investigated as a potential diagnostic tool for TB in high TB-prevalence settings.^34^ The significance of an accelerated BCG reaction in low-TB prevalence countries warrants further study.

Our study is the first to compare injection site reactions between BCG-naïve and BCG-revaccinated adults. Two previous smaller studies have reported similar proportions of injection site reactions (93%–98%) in BCG-revaccinated adults; one study was in 500 HCW in South Africa revaccinated with BCG-Denmark,^35^ and the other was in 64 HCW in Brazil revaccinated with BCG-Moscow.^36^ Neither study reported rates of injection site abscess or lymphadenopathy.

The most common adverse events of special interest in our study were abscess (incidence 1.6%) and regional lymphadenopathy (incidence 3.0%). The incidence of injection site abscess is consistent with studies of BCG-Denmark (1.3%–2.5%) in infants^37,38^ and one Australian study where half of the participants were adults vaccinated with BCG-Connaught.^39^ Within the BRACE trial, fewer injection site abscesses were reported in Stage 2 BCG recipients compared with Stage 1 BCG recipients. The reason for this difference is uncertain; potential contributing factors include differences in vaccinator experience (incorrect administration technique has been associated with abscess formation),^40^ variations between BCG vaccine batches,^41^ co-administration of influenza vaccine in Stage 1, and differences in BCG injection site reaction perceptions by participants in distinct geographical/cultural regions leading to different thresholds for self-reporting concerns regarding injection site. In Stage 2, the majority of participants were recruited in Brazil, which has an active neonatal BCG immunization program and higher TB prevalence, whereas Stage 1 recruitment occurred in Australia, where BCG is not routinely given (approximately half of participants in Stage 1 were BCG naïve) and TB prevalence is low.

Characteristics of BCG injection site abscesses in both trial stages were similar and most (80%) healed within 1 month without medical intervention. Various treatment strategies (antibiotics with and without fine needle aspiration or surgery) were used in the minority of participants who sought external medical advice, reflective of the paucity of robust evidence for the best management approach.^42^

The incidence of BCG-associated lymphadenopathy in our study was also consistent with large studies of BCG-Denmark reporting incidence of non-suppurative lymphadenopathy in children and adolescents (range <1% to 4.8%).^2,30,37,38,43^ Diverse definitions exist in the literature, with a variety of surveillance methods, and using different BCG strains.^10^ We used a broad definition with active surveillance and a higher (adult) BCG dose compared with studies in children.^15^ Moreover, 0.5% of the placebo group reported regional lymphadenopathy, with a similar time to onset, resolution and size to those who received BCG, highlighting that regional lymphadenopathy following vaccination may not always be related to the vaccine itself.

The incidence of urticarial reaction (0.03% (1/3379)) is within range of the reported rate of this rare event (≥1/10000 to <1/1000).^24^ The incidence of keloid scarring (0.06% (2/3379)) within the study period, distinct to the normal BCG scar response, was also within expected range (0.02% to 4.7%).^44^ Future studies could investigate any association with skin pigmentation type.

All SAE were hospital presentations deemed unrelated to vaccination, except for one SUSAR (causal relationship assigned as ‘probable’) and one participant hospitalized with Crohn’s disease (causal relationship assigned as ‘unlikely’). Importantly, there were no disseminated BCG infection or BCG osteitis/osteomyelitis cases (rare severe complications, seen in infants with undiagnosed immunodeficiency).

BCG vaccination during pregnancy is generally not recommended.^45^ To our knowledge, there are no reports on outcomes in women inadvertently vaccinated with BCG during pregnancy. The pregnant woman in our trial who was BCG vaccinated at 2 weeks gestation went on to deliver a healthy infant at term.

Our study has several limitations. First, prior BCG vaccination classification relied on participant self-report without vaccination record confirmation and no information on BCG strain (most participants had their primary BCG vaccination in childhood). However, the majority who reported having received prior BCG also had scar evidence (data not shown). Second, even though the same BCG strain was used for all sites in this study, the vaccinators differed. Vaccine administration technique has been previously shown to influence the incidence of AEFI.^46^ Although all BRACE trial vaccinators were trained in intradermal BCG delivery, with an emphasis on the presence of a post-injection wheal as a marker of correct delivery, intradermal vaccination is a challenging technique to master.^23^ Third, the injection site itch question was added only after many participants commented on intense itch at the start of the trial, and recall bias cannot be excluded.

Strengths of our study include the active safety surveillance of over 5000 healthcare workers across three continents comprising both high and low TB-prevalence settings, using standard case definitions for adverse events, with safety data available for 99% participants. Safety medical doctors met regularly to discuss AE as they occurred, reaching consensus decisions on classification.

In conclusion, BCG revaccination was found to be safe and well tolerated in adults. Although local injection site reactions were more common in BCG-revaccinated than in BCG-naïve adults, these were usually self-limited and the duration of erythema and swelling was shorter. We found itch at the BCG injection site to be a common initial symptom and recommend its addition to the list of expected BCG reactions. Injection site abscess and regional lymphadenopathy, the most common significant adverse events, had a benign course and self-resolved within a month in most participants. This study shows that BCG vaccination and revaccination has an acceptable safety profile in adults at a time when BCG is being increasingly considered for its novel indications.

## Supplementary Material

Supplemental Material

Supplemental Material

## Data Availability

Deidentified participant data and data dictionary are available to others on request and on completion of a signed data access agreement. Requests can be made in writing to braceresearch@mcri.edu.au.
