# Supplementary material for: Safety of BCG vaccination and revaccination in healthcare workers
Source: Hum Vaccin Immunother. 2023 Aug 8;19(2):2239088. doi: 10.1080/21645515.2023.2239088 (PMC10411308; doi:10.1080/21645515.2023.2239088)
Supplement: Supplemental Material [file KHVI_A_2239088_SM2767.pdf]

Supplementary Table 1.

Local injection site reaction toxicity grading scale

Local reaction	Grade 0<br>None	Grade 1<br>Mild	Grade 2<br>Moderate	Grade 3<br>Severe	Grade 4<br>Potentially life-threatening
**Pain**	None	Does not interfere with activity	Repeated use of non-narcotic pain reliever > 24 hours or interferes with activity	Any use of narcotic pain reliever or prevents daily activity	Emergency room visit or hospitalisation
**Tenderness**	None	Mild discomfort to touch	Discomfort with movement	Significant discomfort at rest	Emergency room visit or hospitalization
**Erythema/redness**	None	2.5 - 5 cm	5.1 - 10 cm	>10 cm	Necrosis or exfoliative dermatitis
**Swelling / induration**	None	2.5 - 5 cm and does not interfere with activity	5.1 - 10 cm or interferes with activity	>10 cm or prevents daily activity	Necrosis
**Itch**	None	Itching localised to injection site that is relieved spontaneously or in <48 hours of treatment	Itching beyond the injection site that is not generalised OR Itching localised to injection site requiring ≥48 hours of treatment	Generalised itching causing inability to perform usual social & functional activities	Not applicable

Injection site pain, tenderness, erythema and swelling, as per Food and Drug Administration. (2007). "Guidance for Industry: toxicity grading scale for healthy adult and adolescent volunteers enrolled in preventive vaccine clinical". Available from https://www.fda.gov/downloads/BiologicsBloodVaccines/GuidanceComplianceRegulatoryInformation/Guidances/Vaccines/ucm091977.pdf.
Injection site itch as per U.S. Department of Health and Human Services, National Institutes of Health, National Institute of Allergy and Infectious Diseases, Division of AIDS. Division of AIDS (DAIDS) (2017). “Table for Grading the Severity of Adult and Pediatric Adverse Events, Corrected Version 2.1”. Available from: https://rsc.niaid.nih.gov/sites/default/files/daidsgradingcorrectedv21.pdf

Supplementary Table 2. Serious adverse events (SAE)

BRACE Stage 1

Participant	Sex	Vaccine group	Onset following vaccination	Description of adverse event	SAE type[a]	Treatment	Relationship to vaccine[b]<br><br>Site Investigator (S) assessment<br><br>MCRI Sponsor (M) assessment	Severity of SAE[c]	Outcome
1	F	Influenza+BCG	8 hours	Sudden onset severe headache ('worst headache in life') then myalgia, vomiting, fever 38.3°C, tachycardia. COVID-19 testing negative. Taken to hospital via ambulance.	3	Hospitalisation overnight: Intravenous paracetamol, ondansetron, metoclopramide, fluid therapy.	(S)(M) Possibly related to influenza vaccine	Severe	Discharged home Day 2
2	F	Influenza+BCG	10 hours	Developed sore throat and felt unwell on evening of Day 1. Day 2 woke up with sore throat, wheeze and cough. Known past medical history of mild asthma. Used salbutamol inhaler at home, with limited effect. COVID-19 testing negative. Worsening cough and wheeze over next few hours led to emergency department (ED) presentation. In ED, afebrile, no sign of respiratory distress or audible wheeze on auscultation. Mild erythematous pharynx.	7	Emergency department presentation: salbutamol nebuliser and 25mg of oral prednisolone. Discharged home within two hours.	(S)(M) Possibly related to influenza vaccine in the context of past medical history of previous mild asthma.	Severe	Discharged home Day 2
3	F	Influenza+BCG	Day 58	Overnight hospitalisation for an episode of pyelonephritis, on a background of chronic urological health issues (long term stoma and indwelling catheter). Frequent urinary tract infections.	3	Hospitalisation overnight: 24 hours of intravenous antibiotics and intravenous fluid therapy.	(S)(M) Unrelated	Severe	Discharged home Day 59
4	F	Influenza+BCG	Day 68	Hospitalisation for shoulder surgery secondary to workplace accident.	3	Hospitalisation: shoulder surgery	(S)(M) Unrelated	Severe	Discharged home Day 70
5	F	Influenza+BCG	Day 71	5-night hospitalisation for abdominal pain with history of diverticulitis. Diagnosed with diverticulitis.	3	Hospitalisation: antibiotics and analgesia	(S)(M) Unrelated	Severe	Discharged home Day 77
6	F	Influenza+BCG	Day 63	Hospitalisation for unmanageable back pain and altered sensation in left foot. MRI spine diagnosis of L5 & S1 nerve impingement and L4, L5, S1 disc prolapse.<br>Past history of fractured back in childhood.	3	Hospitalisation: CT-guided nerve block, analgesia and supportive care. Insertion of indwelling catheter	(S)(M) Unrelated	Severe	Discharged home Day 71
7	F	Influenza+BCG	Day 23	2-night hospitalisation for acute appendicitis	3	Hospitalisation: laparoscopic appendectomy	(S) Unlikely<br>(M) Unrelated	Severe	Discharged home Day 25
8	F	Influenza+BCG	Day 23	Hospitalisation for mental health concerns.	3	Hospitalisation	(S)(M) Unrelated	Severe	Discharged home Day 26
9	F	Influenza+BCG	Day 17	Hospitalisation for hand/wrist cellulitis; infected cat bites, multiple puncture marks.	3	Hospitalisation: surgical washout and debridement of injured right hand/wrist and antibiotics.	(S)(M) Unrelated	Severe	Discharged home Day 19
10	F	Influenza	Day 20	Overnight hospitalisation for infected sebaceous cyst on neck.	3	Hospitalisation: drainage of cyst and intravenous antibiotics	(S)(M) Unrelated	Severe	Discharged home Day 21

11	F	Influenza	Day 27	4-day hospitalisation for fever, sore throat, headaches, photophobia. Investigated for COVID-19 (negative), blood cultures, urine, brain imaging, lumbar puncture. Diagnosis: tonsillitis	3	Hospitalisation: intravenous fluid therapy for 3 days, antibiotics and antivirals. Regular analgesia	(S)(M) Unrelated	Severe	Discharged home Day 33
12	F	Influenza	Day 64	Hospitalisation for two nights for fractured ankle requiring internal fixation.	3	Hospitalisation: surgery for fractured ankle requiring internal fixation.	(S)(M) Unrelated	Severe	Discharged home Day 66
13	M	Influenza	Day 80	Hospitalisation for sports injury; lower limb fractures requiring surgery.	3	Hospitalisation: surgery for lower limb fractures.	(S)(M) Unrelated	Severe	Discharged home Day 82
14	F	Influenza	Day 83	5-day hospitalisation for mental health concerns. Tested negative twice for COVID-19 during hospital stay (respiratory symptoms).	3	Hospitalisation	(S)(M) Unrelated	Severe	Discharged home Day 88
15	M	Influenza	Day 56	2-night hospitalisation for lower abdominal pain due to diverticulitis.	3	Hospitalisation: antibiotics and analgesia	(S) Unlikely<br>(M) Unrelated	Severe	Discharged home Day 58
16	F	Influenza	Day 14	Hospitalisation for investigation for iron deficiency and possible inflammatory bowel syndrome; During hospitalisation, fell and hit head, with subsequent seizure. Found to have hyponatraemia, Past history of childhood epilepsy.	3	Hospitalisation: seizure investigations, electrolyte abnormality corrected, prophylactic antibiotics.	(S)(M) Unrelated	Severe	Discharged home Day 17

BRACE Stage 2

Participant	Sex	Vaccine group	Onset following vaccination	Description of adverse event	SAE type[a]	Treatment	Relationship to vaccine[b]<br><br>Site Investigator (S) assessment<br><br>MCRI Sponsor (M) assessment	Severity of SAE[c]	Outcome
1	F	BCG	Day 21	Hospitalisation for injection site abscess with pus discharge (approx. 80ml) and systemic symptoms. Plastics team review on readmission and immunology assessment.	3	Hospitalisation: IV flucloxacillin and gentamicin, IV fluids	(S)(M) Probable	Severe	Discharged home Day 37
2	F	BCG	Day 78	Overnight hospitalisation in emergency department for Crohn's disease	3	Hospitalisation: faecal calprotectin test. Crohn's disease diagnosed on subsequent colonoscopy	(S)(M) Unlikely	Severe	Discharged home Day 79
3	F	BCG	Day 29	Hospitalisation for cellulitis secondary to a cat scratch on right hand. No associated lymphadenopathy or fever. Injection site reaction healing well.	3	Hospitalisation: IV antibiotics	(S)(M) Unrelated	Severe	Discharged home Day 31
4	F	BCG	Day 5	Discovered lump in right breast; referred to breast specialist; biopsy diagnosis of breast cancer.	7	Chemotherapy	(S)(M) Unrelated	Severe	Withdrawal from study on Day 12
5	M	BCG	Day 24	Hospitalisation for acute vomiting, diarrhoea and dehydration.	3	Hospitalisation: hydration	(S)(M) Unrelated	Severe	Discharged home Day 25
6	M	BCG	Day 43	Hospitalisation for COVID-19; mechanical ventilation in intensive care unit.	2	Hospitalisation: mechanical ventilation	(S)(M) Unrelated	Life-threatening	Discharged home Day 139

7	F	BCG	Day 91	Hospitalisation for COVID-19 pneumonia.	3	Hospitalisation	(S)(M) Unrelated	Severe	Discharged home Day 96
8	M	BCG	Day 68	Hospitalisation for acute appendicitis.	3	Hospitalisation	(S)(M) Unrelated	Severe	Discharged home Day 69
9	M	BCG	Day 46	Hospitalisation for COVID-19; mechanical ventilation in intensive care unit.	2	Hospitalisation: mechanical ventilation	(S)(M) Unrelated	Life-threatening	Discharged home Day 64
10	F	BCG	Day 65	Hospitalised for cardiac symptoms, related to underlying chronic disease.	3	Hospitalisation	(S)(M) Unrelated	Severe	Discharged home Day 68
11	F	BCG	Day 73	Hospitalisation for COVID-19; presented with shortness of breath and decompensated diabetes (ketoacidosis).	2	Hospitalisation: intensive care unit	(S)(M) Unrelated	Life-threatening	Discharged home Day 82
12	M	BCG	Day 54	First hospitalisation: overnight admission to intensive care unit following planned surgery for transurethral resection of the prostate and removal of wires from previously fractured patella.	4	Hospitalisation: intensive care unit, cardiac echocardiogram, right leg Doppler ultrasound.	(S)(M) Unrelated	Life-threatening	Discharged home Day 79
13	M	BCG	Day 80	Second hospitalisation for fever and haematuria following transurethral resection of the prostate.	3	Hospitalisation: urinary tract ultrasound, septic work-up.	(S)(M) Unrelated	Severe	Discharged home Day 84
14	F	BCG	Day 91	Hospitalisation for cough and shortness of breath; COVID-19 negative.	2	Hospitalisation	(S)(M) Unrelated	Severe	Discharged home Day 94
15	M	BCG	Day 86	Hospitalisation for appendicitis, with emergency surgery.	3	Hospitalisation: emergency surgery	(S)(M) Unrelated	Severe	Discharged home Day 88
16	F	BCG	Day 37	Hospitalisation for epigastric pain.	3	Hospitalisation: symptomatic treatment	(S)(M) Unrelated	Severe	Discharged home Day 40
17	F	BCG	Day 20	Hospitalisation for abdominal pain; gallstone blocking bile duct.	3	Hospitalisation: surgery to remove gallstones	(S)(M) Unrelated	Severe	Discharged home Day 25
18	F	BCG	Day 89	Prolonged hospitalisation due to complication following endoscopic submucosal dissection for removal of a rectal carcinoma.	3	Hospitalisation: surgery for removal of carcinoma. No chemotherapy or radiotherapy requirement.	(S)(M) Unrelated	Severe	Discharged home on Day 91
19	F	BCG	Day 88	Hospitalisation for renal infection.	2	Hospitalisation	(S)(M) Unrelated	Severe	Discharged home Day 91
20	F	BCG	Day 63	Hospitalisation for psychiatric illness – depression.	2	Hospitalisation	(S)(M) Unrelated	Life-threatening	Discharged home Day 77
21	F	BCG	Day 4	Hospitalisation for vascular catheterisation; participant fell in pool and damaged portacath. Portacath had been inserted three years prior during management of breast cancer.	2	Hospitalisation: vascular catheterisation for removal of damaged portacath	(S)(M) Unrelated	Severe	Discharged home Day 5
22	F	Placebo	Day 25	Hospitalisation for COVID-19; mechanical ventilation in intensive care unit.	2	Hospitalisation: mechanical ventilation	(S)(M) Unrelated	Death	Death on Day 49
23	M	Placebo	Day 21	Hospitalisation for COVID-19.	2	Hospitalisation: non-invasive ventilation	(S)(M) Unrelated	Life-threatening	Discharged home Day 28
24	F	Placebo	Day 28	Hospitalisation for COVID-19.	2	Hospitalisation: non-invasive ventilation	(S)(M) Unrelated	Life-threatening	Discharged home Day 31
25	F	Placebo	Day 90	Hospitalisation for ankle fracture injury secondary to being run-over.	3	Hospitalisation: ankle surgery (right tibia)	(S)(M) Unrelated	Severe	Discharged home Day 96

26	F	Placebo	Day 72	Hospitalisation for femur fracture secondary to skate fall.	3	Hospitalisation: hip surgery (left femur)	(S)(M) Unrelated	Severe	Discharged home Day 78
27	M	Placebo	Day 39	Hospitalisation for myocardial infarction.	3	Hospitalisation	(S)(M) Unrelated	Severe	Discharged home Day 42
28	M	Placebo	Day 82	Hospitalisation for dengue with complications.	2	Hospitalisation	(S)(M) Unrelated	Life-threatening	Discharged home Day 85
29	M	Placebo	Day 86	Hospitalisation for COVID-19; shortness of breath presentation.	2	Hospitalisation	(S)(M) Unrelated	Life-threatening	Discharged home Day 101
30	F	Placebo	Day 15	Hospitalisation for blunt cut injury to left hand (with left index finger paraesthesia) secondary to domestic knife incident.	3	Hospitalisation: exploratory surgery for neurological damage and carpal tunnel release.	(S)(M) Unrelated	Severe	Discharged home Day 16

Note: day 1= day of vaccination

[a] **SAE type = criteria for seriousness**
1. Resulted in death
2. Immediately life-threatening
3. Requires inpatient hospitalisation (i.e. minimum overnight admission that is non-elective).
4. Results in prolongation of existing hospitalisation
5. Results in persistent or significant disability/incapacity
6. Is a congenital anomaly/birth defect
7. In the medical judgment of the treating physician and/or investigator, it may jeopardise the participant or require intervention to prevent one of the above outcomes

[b] **Severity of SAE:**
- Severe (severe medically significant but not immediately life threatening)
- Life-threatening (immediately life-threatening)
- Death related to adverse event

[c] **Definition of relationship to the intervention**
- Unrelated (The AE is clearly NOT related to intervention)
- Unlikely (The AE is doubtfully related to the intervention)
- Possible (The AE may be related to the intervention)
- Probable (The AE is likely related to the intervention)
- Definite (The AE is clearly related to the intervention)

Supplementary Table 3. Vaccination during pregnancy

Participant	Vaccine	Country	Age (years) at vaccination	BMI (kg/m^2)	Co-morbidity	Smoker	GA at vaccination	GA at delivery	Pregnancy outcome	Congenital anomaly or birth defect
1	BCG	Brazil	36	28	No	No	2 weeks + 1 day	37 weeks	Baby born alive and healthy	No
2	Placebo	Brazil	30	41	No	Yes	7 weeks + 1 day	40 weeks	Baby born alive and healthy	No
3	Placebo	Brazil	34	25	No	No	3 weeks + 6 days	39 weeks	Baby born alive and healthy	No
4	Placebo	Brazil	38	23	No	No	2 weeks	40 weeks	Baby born alive and healthy	No
