# Supplementary material for: Safety of BCG vaccination and revaccination in healthcare workers
Source: Hum Vaccin Immunother. 2023 Aug 8;19(2):2239088. doi: 10.1080/21645515.2023.2239088 (PMC10411308; doi:10.1080/21645515.2023.2239088)
Supplement: Supplemental Material [file KHVI_A_2239088_SM2763.pdf]

Case definitions

BCG injection site abscess was defined as a localised collection of pus, ≥1.5 cm in diameter at the injection site. BCG-associated lymphadenopathy was defined as palpable regional (ipsilateral axilla or neck) lymph node enlargement. Participants reporting lymphadenopathy ≥1.5 cm in diameter or persistent (>2 weeks duration) were recommended to seek medical assessment.

An SAE was defined as an adverse event that resulted in death, was immediately life-threatening, required hospitalisation or prolongation of existing hospitalisation, resulted in persistent or significant disability/incapacity, or was a congenital anomaly/birth defect, regardless of causal relation to vaccination. Elective surgery or other elective hospitalisation were not considered SAE.
